# Verbal abilities in children of mothers with epilepsy: Association to maternal folate status

**DOI:** 10.1212/WNL.0000000000006073

**Published:** 2018-08-28

**Authors:** Elisabeth Synnøve Nilsen Husebye, Nils Erik Gilhus, Bettina Riedel, Olav Spigset, Anne Kjersti Daltveit, Marte Helene Bjørk

**Affiliations:** From the Department of Clinical Medicine (E.S.N.H., N.E.G., M.H.B.), Section for Neurology, Department of Clinical Science (B.R.), and Department of Global Public Health and Primary Care (A.K.D.), University of Bergen; Department of Neurology (E.S.N.H., N.E.G., M.H.B.) and Laboratory of Clinical Biochemistry (B.R.), Section of Clinical Pharmacology, Haukeland University Hospital, Bergen; Department of Clinical Pharmacology (O.S.), St. Olav University Hospital; Department of Clinical and Molecular Medicine (O.S.), Norwegian University of Science and Technology, Trondheim; and Department of Health Registries (A.K.D.), Norwegian Institute of Public Health, Bergen, Norway.

## Abstract

**Objective:**

To examine the effect of maternal folic acid supplementation and maternal plasma folate and antiepileptic drug (AED) concentrations on language delay in AED-exposed children of mothers with epilepsy.

**Methods:**

Children of mothers with and without epilepsy enrolled from 1999 to 2008 in the Norwegian Mother and Child Cohort study were included. Information on medical history, AED use, and folic acid supplementation during pregnancy was collected from parent-completed questionnaires. Maternal plasma folate and maternal plasma and umbilical cord AED concentrations were measured in blood samples from gestational weeks 17 to 19 and immediately after birth, respectively. Language development at 18 and 36 months was evaluated by the Ages and Stages Questionnaires.

**Results:**

A total of 335 AED-exposed children of mothers with epilepsy and 104,222 children of mothers without epilepsy were surveyed. For those with no maternal periconceptional folic acid supplementation, the fully adjusted odds ratio (OR) for language delay in AED-exposed children compared to the controls at 18 months was 3.9 (95% confidence interval [CI] 1.9–7.8, *p* < 0.001) and at 36 months was 4.7 (95% CI 2.0–10.6, *p* < 0.001). When folic supplementation was used, the corresponding ORs for language delay were 1.7 (95% CI 1.2–2.6, *p* = 0.01) and 1.7 (95% CI 0.9–3.2, *p* = 0.13), respectively. The positive effect of folic acid supplement use on language delay in AED-exposed children was significant only when supplement was used in the period from 4 weeks before the pregnancy and until the end of the first trimester.

**Conclusion:**

Folic acid use early in pregnancy may have a preventive effect on language delay associated with in utero AED exposure.

Most women with epilepsy are dependent on treatment with antiepileptic drugs (AEDs) throughout their pregnancy to prevent epileptic seizures.^1^ AEDs increase the risk of congenital malformations in a dose-dependent manner.^2^ Some AEDs have also been associated with impaired neurodevelopment and behavioral disorders in the offspring.^1,2^ Hence, it is crucial to identify factors that modulate the risk of AED-related fetal harm.

Folate is a B vitamin important for normal brain development.^3^ Many AEDs interact with folate metabolism and have been associated with reduced plasma folate.^4,5^ There is growing evidence of a positive association between maternal folate status during pregnancy and neurodevelopmental outcome in the offspring.^3,6–8^ Few studies have examined whether folic acid supplementation protects against impaired neurodevelopment after AED exposure in utero. Some studies have indicated that folic acid may have a positive effect on IQ and verbal abilities in children exposed to AEDs in utero,^9,10^ but the results are conflicting.^11,12^ We have previously found that AED-exposed children have fewer autistic traits if their mothers used folic acid supplements in the periconceptional period.^13^ Women in Norway are recommended to use 0.4 mg folic acid daily in the periconceptional period only, while women with epilepsy who use AEDs usually are recommended to use 1 to 5 mg daily in the periconceptional period and 0.4 mg daily in the second and third trimesters. There is no mandatory folic acid food fortification in Norway.^14^

The aim of our study was to investigate the effect of maternal folic acid supplement use, maternal plasma folate, and AED concentrations during pregnancy on language development in AED-exposed children of mothers with epilepsy.

## Methods

### Study population

The study population consisted of women and children included in the Norwegian Mother and Child Cohort Study (MoBa). MoBa is a prospective, ongoing population-based pregnancy cohort study conducted by the Norwegian Institute of Public Health and is linked to the compulsory Medical Birth Registry of Norway (MBRN).^15^ Norwegian-speaking women were invited to participate from 1999 to 2008. The participation rate was 41%. Information on background, medical history, medication use, vitamin and folic acid intake, and child development, including language function, was obtained by parent-completed questionnaires. The questionnaires were answered in gestational weeks 17 to 19 (Q1) and 30 (Q2) and when the child was 18 and 36 months old (Q3 and Q4; response rates, 72% and 56%, respectively). Maternal blood samples were collected at week 17 to 19 of gestation and from the umbilical cord immediately after delivery.

The epilepsy diagnosis is based on self-reported information from the MoBa questionnaires and information from the MBRN registered by the family doctor or midwife.^16^ We have previously validated the epilepsy cohort in MoBa (data available from Dryad, Methods, doi.org/10.5061/dryad.1237b6m), and the validity was very good.^17^

**Figure 1:**
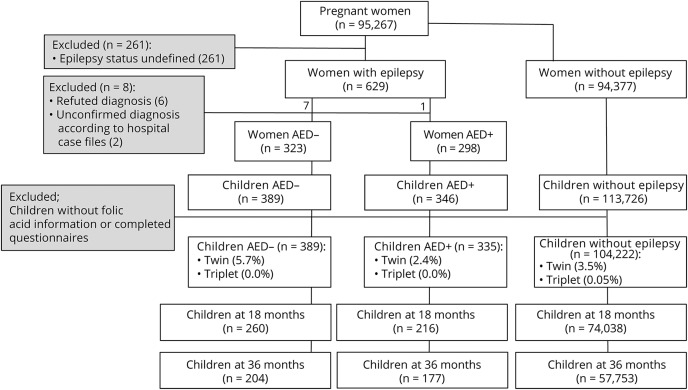
Flowchart of excluded and included cases AED+ = antiepileptic drug use/exposure; AED− = no antiepileptic drug use/exposure.

Our material is based on version VIII of the MoBa databank and consisted of 724 children of 616 mothers with epilepsy and 104,222 children of 86,443 mothers without epilepsy with available information on maternal folic acid supplement use during pregnancy (figure 1). The children of mothers with epilepsy were further classified into 2 groups: 1 group exposed to AEDs in utero (n = 335), our main study group, and another group not exposed to AEDs in utero (n = 389). We have previously reported on general development after in utero and breastmilk exposure to AED in this cohort.^18,19^

### Variables

#### Maternal folic acid supplementation

Intake of folic acid before and during pregnancy was reported in gestational week 17 to 19 (Q1) for the following time intervals: >5 weeks before pregnancy, 4 weeks before pregnancy (preconception), and use during gestational weeks 0 to 4, 5 to 8, 9 to 12, and 13+. Folic acid use in gestational weeks 13 to 16, 17 to 20, 21 to 24, 25 to 29, and 29+ was reported in gestational week 30 (Q2) (data available from Dryad, Methods, doi.org/10.5061/dryad.1237b6m). Folic acid doses were obtained for 139 AED-exposed children and 160 AED-unexposed children by a separate retrospective questionnaire to women with epilepsy in our previous validation study (Q5; response rate 50%).^17^ In 84 children (25%) in the AED-exposed group and 21 children (5%) in the AED-unexposed group, the mothers reported a daily intake of folic acid of ≥1 mg. We defined periconceptional folic acid use as maternal intake of folic acid supplements from 4 weeks before the start of the pregnancy and/or during the first trimester.

#### AED use

Information on AED use and type of medication was collected from self-reported information in Q1 and the MBRN data registered by the family doctor or midwife.^15^ There was 100% agreement between self-reported AED use in MoBa and the reported AED use in hospital records in our previous validation study.^17^

#### Measurement of plasma folate and AED concentrations

From the MoBa biobank,^20^ folate was available in maternal plasma samples obtained at gestational week 17 to 19 for 228 AED-exposed children (68%). Analysis included the biologically active 5-methyltetrahydrofolate (mTHF) and the degradation product 4-alfa-hydroxy-5-methyltetrahydrofolate (hmTHF). mTHF represents the prevailing folate form in plasma. This form is unstable in blood samples kept at room temperature, but is largely recovered as hmTHF. Hence, maternal plasma folate is given as the sum of the concentration of mTHF and hmTHF.^21,22^

The concentrations of valproate, lamotrigine, carbamazepine, carbamazepine10,11-epoxide, levetiracetam, topiramate, and the oxcarbazepine monohydroxy derivative metabolite were analyzed in 226 maternal plasma samples obtained at gestational week 17 to 19 and in 198 samples from the umbilical cord, as described previously,^17^ for a total of 255 AED-exposed children (76%). In 238 of these samples (93%), the reported AED was detected. For the statistical analysis, the plasma concentrations were normalized relative to the ranges observed within each group according to the following formula: 100 × (observed concentration − minimum concentration)/concentration range.^10,23^ The mean of the normalized plasma concentrations was calculated for each child on the basis of both the concentration from the maternal sample and the umbilical cord sample if both were present. If only one of the samples was available, this concentration was used. If a child was exposed to AED polytherapy, the mean normalized concentrations of each AED were added together.

#### Language delay

##### Global language delay

In Q3 and Q4, mothers completed a 3-item and a 6-item version, respectively (data available from Dryad, table 1, doi.org/10.5061/dryad.1237b6m), of the 18 months' and 36 months' communication scale from the Ages and Stages Questionnaires (ASQ).^24^ ASQ is considered a reliable screening tool with high concurrent validity.^24,25^ Each item had the following answer options: yes (10 points), sometimes (5 points), and not yet (0 points). The maximum score reflecting no language delay was 30 and 60 points at 18 and 36 months, respectively. Children with missing answers in Q3 were excluded. If only 1 answer was missing in Q4, this was imputed with the estimation-maximization procedure in SPSS (IBM, Armonk, NY). Children were defined as having global language delay when the mothers had reported an ASQ score >1.5 SD below the mean ASQ score in the total MoBa cohort.^24,26^

##### Expressive language delay

In Q4, a 1-item question regarding expressive language skills has shown acceptable validity as an indicator of the grammatical complexity level of 3-year-old children (data available from Dryad, table 2, doi.org/10.5061/dryad.1237b6m).^27^ The maximum score reflecting no expressive language delay was 6 points. Children talking in 2- to 3-word phrases or less were classified as having expressive language delay.

### Covariates

Relevant covariates were selected from the MoBa questionnaires and from the MBRN^6,28^: parental higher education (≥17 years of schooling), maternal low education (≤9 years of schooling), total household income <400,000 Norwegian kroner annually (equals approximately €42,000), unplanned pregnancy, smoking and alcohol use (consumption ≥1 per month) in pregnancy, parity (number of previous pregnancies with >21 gestation weeks), maternal age, maternal depression and anxiety symptoms during pregnancy (mean score >1.75 on the Hopkins symptom checklist^29^ at gestational week 17–19), single mother, maternal prepregnancy body mass index, seizures during pregnancy, tonic-clonic seizures during pregnancy (data available from Dryad, Methods, doi.org/10.5061/dryad.1237b6m), AED polytherapy, twin or triplet children, Apgar score 5 minutes after birth, gestational age, and offspring sex.

### Statistical analysis

The statistical analysis was performed with IBM SPSS software version 24. AED-exposed and -unexposed children of mothers with epilepsy were compared to a control group of children of mothers without epilepsy. Each of the 3 groups was stratified by periconceptional folic acid use. Groups with similar periconceptional folic acid supplementation status were compared. We also compared the supplemented group with the unsupplemented group within each of the 3 groups. Categorical variables were compared with the χ^2^ test for independence or Fisher exact test when appropriate. Continuous variables were compared with the Mann-Whitney *U* test because of violation of the assumption of normal distribution. The risk for delayed language outcome was investigated with logistic regression. The relationship between maternal plasma folate status/AED concentrations and language outcome was examined by a multivariable linear regression model and by correlation analysis. Values of *p* < 0.05 were considered statistically significant. We hypothesized a causal relationship between no periconceptional folic acid supplementation and language delay to calculate the attributable risk (AR) of no periconceptional folic acid supplementation on language delay in each of the 3 groups (data available from Dryad, Methods, doi.org/10.5061/dryad.1237b6m). This was done by calculating relative risk (RR) in a 2 × 2 table and then the AR with the formula AR = RR − 1/RR.^30^

### Standard protocol approval, registration, and patient consent

The establishment and data collection in MoBa obtained a license from the Norwegian Data Inspectorate and approval from the Regional Committee for Medical Research Ethics.

The current study was approved by the Regional Committee for Medical Research Ethics (reference No. 2011/1616). Written informed consent was obtained from all participating parents in MoBa.

### Data availability

Data from MoBa and the MBRN used in this study are managed by the national health register holders in Norway and can be made available to researchers, provided that necessary approval is obtained from the Regional Ethics Committees in Norway and from the data owners. The Norwegian Institute of Public Health has a general contact point for data access at the following e-mail address: datatilgang@fhi.no.

## Results

Characteristics of the children, their parents, and the pregnancies stratified by periconceptional folic acid use are presented in table 1 (full version: data available from Dryad, table 3, doi.org/10.5061/dryad.1237b6m). A total of 268 children were exposed to AED monotherapy in utero, and 65 children were exposed to AED polytherapy (data available from Dryad, table 4). In children exposed to monotherapy, the most frequently used AEDs were lamotrigine (39%), carbamazepine (26%), valproate (15%), levetiracetam (6%), topiramate (4%), and oxcarbazepine (3%). In the polytherapy group, the most frequently used AEDs were lamotrigine (51%), carbamazepine (32%), valproate (29%), levetiracetam (29%), oxcarbazepine (23%), and topiramate (15%). For 2 children, the AED drug regimen was unspecified.

**Table 1:**
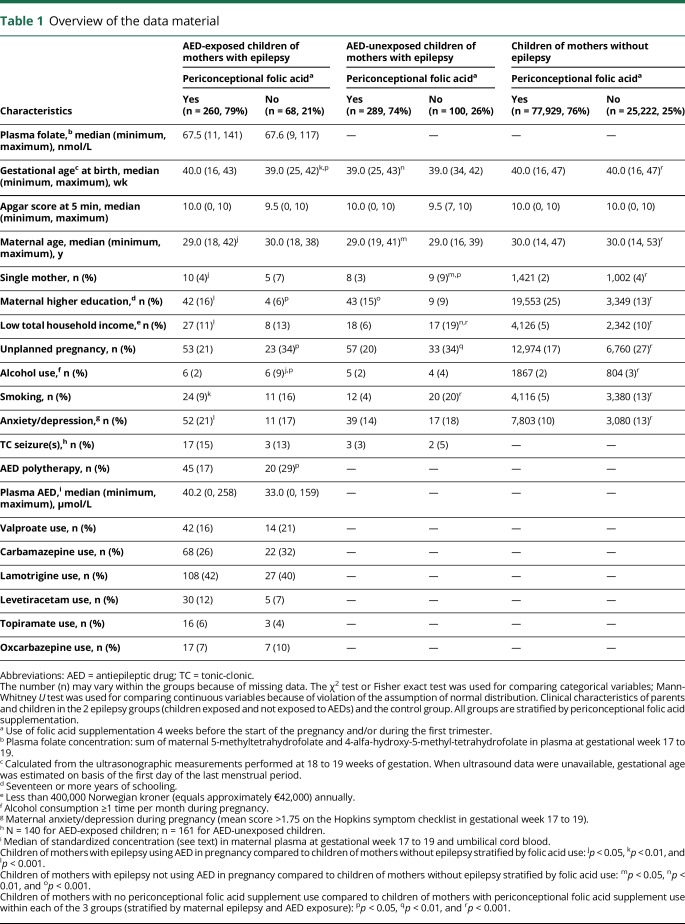
Overview of the data material

### Folic acid supplementation and language delay

Without periconceptional folic acid supplementation, 34% of the AED-exposed children had global language delay at 18 months compared to 11% in the control group without maternal epilepsy (*p* < 0.001) (table 2). The fully adjusted odds ratio (OR) was 3.9 (95% confidence interval [CI] 1.9–7.8, *p* < 0.001) (table 3). At 36 months, 24% of AED-exposed children had expressive language delay compared to only 6% in the control group (*p* < 0.001). The fully adjusted OR was 4.7 (95% CI 2.0–10.6, *p* < 0.001). In the children of mothers who had used folic acid periconceptionally, 17% of AED-exposed children had global language delay at 18 months compared to 11% in the control group (*p* = 0.01). The fully adjusted OR was 1.7 (95% CI 1.2–2.6, *p* = 0.01). For expressive language delay at 36 months with folic acid, 7% of AED-exposed children had a delay compared to 4% in the control group (*p* = 0.08). The fully adjusted OR was 1.7 (95% CI 0.9–3.2, *p* = 0.13). There were no significant differences between AED-unexposed children of mothers with epilepsy and the control group (tables 2 and 3).

**Table 2:**
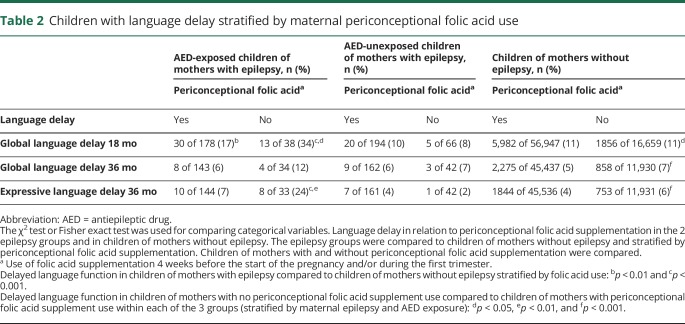
Children with language delay stratified by maternal periconceptional folic acid use

**Table 3:**
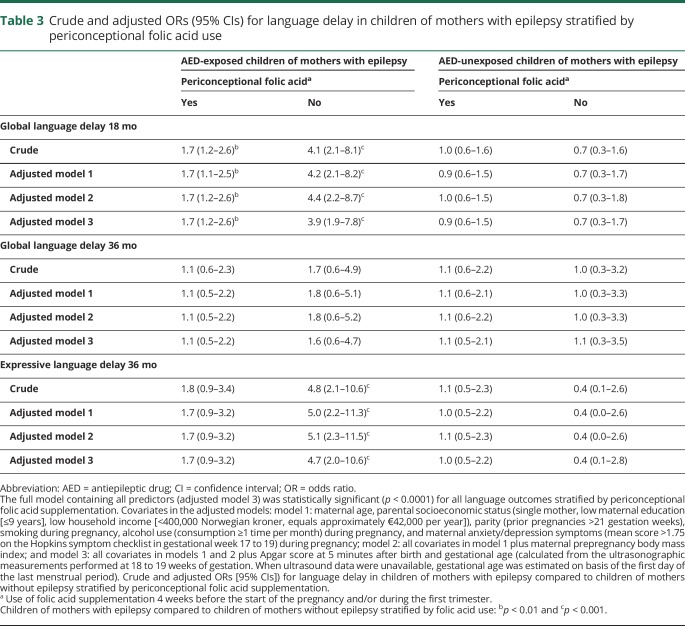
Crude and adjusted ORs (95% CIs) for language delay in children of mothers with epilepsy stratified by periconceptional folic acid use

Within the group of AED-exposed children, the proportion of children with language delay was higher in the no supplementation group than in the supplemented group (table 2). A difference was also found in children of women without epilepsy, but it was much smaller than for the AED-exposed children. Stratification by AED revealed that the number of lamotrigine-exposed children with language delay was significantly higher in the no supplementation group compared to the supplemented group (table 4). The same tendency was seen for children exposed to valproate and carbamazepine, but this was not significant (table 4).

**Table 4:**
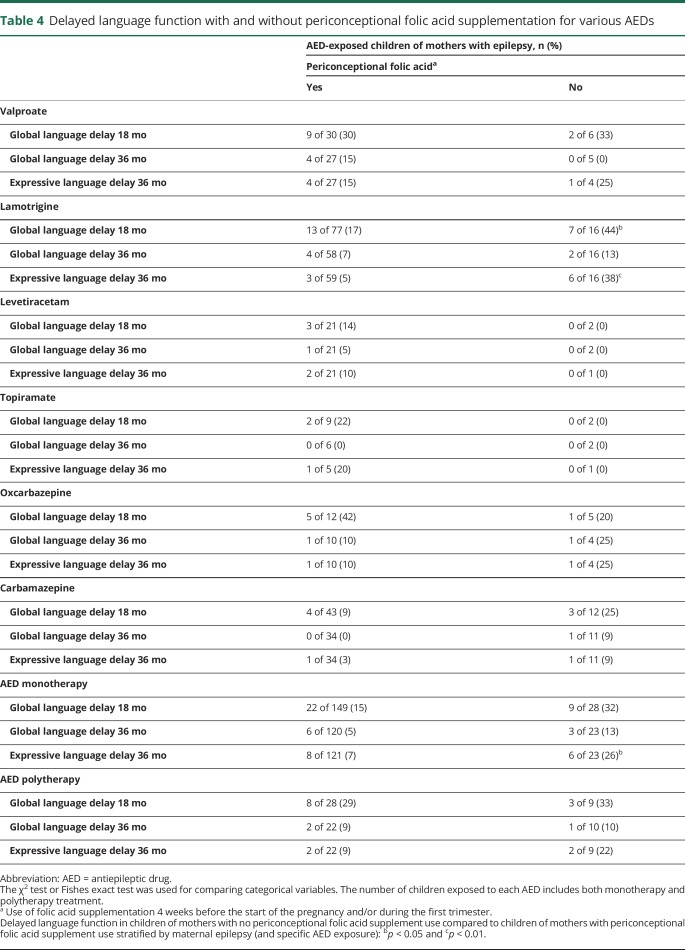
Delayed language function with and without periconceptional folic acid supplementation for various AEDs

**Figure 2:**
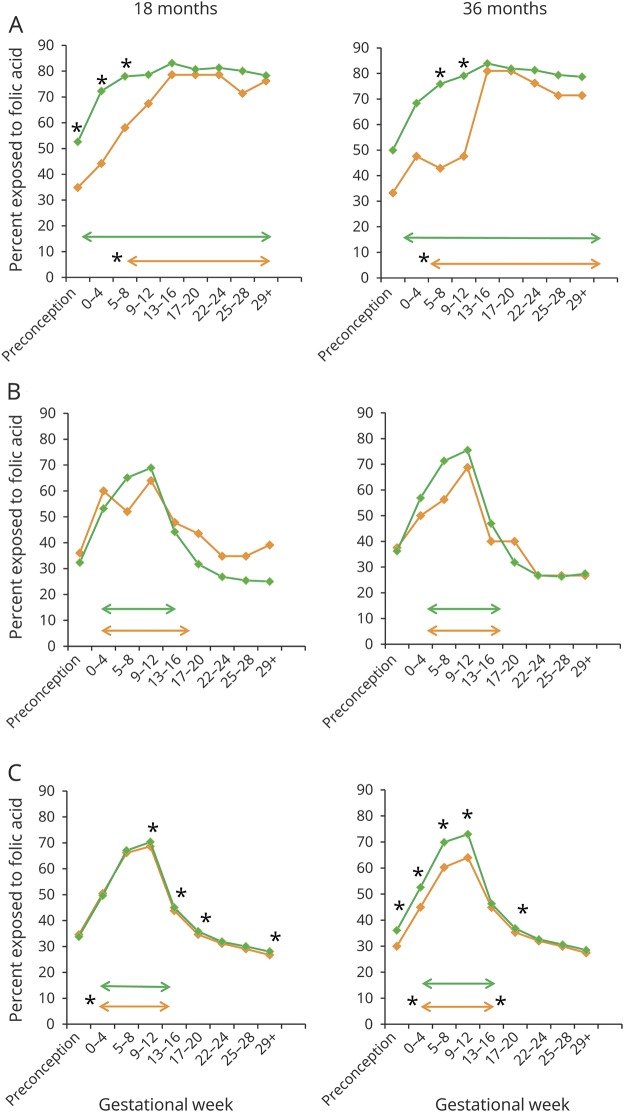
Relationship between language delay and timing of maternal folic acid intake Graphs illustrate the proportion of children (percent) exposed to maternal folic acid supplementation at different time intervals during pregnancy and the relationship to language delay (orange lines) and no language delay (green lines) at 18 and 36 months. Language delay at 36 months includes global language delay and expressive language delay. Arrows illustrate median start and median stop of maternal folic acid supplementation during pregnancy. Significant differences in folic acid supplementation (χ^2^ test for independence) and median start/stop of folic acid supplementation (Mann-Whitney *U* test) are marked with asterisks. (A) Antiepileptic drug (AED)–exposed children of mothers with epilepsy at 18 months (n = 216) and 36 months (n = 179). (B) AED–unexposed children of mothers with epilepsy at 18 months (n = 260) and 36 months (n = 204). (C) Children of mothers without epilepsy at 18 months (n = 73,606) and 36 months (n = 57,715). Statistically significant differences were seen even with minor or no differences in percentages (*p* values between 0.02 and 0.05) or medians because of a high number of observations.

Mothers of AED-exposed children with language delay started with folic acid later in pregnancy. The median start of folic acid supplementation was gestational week 6.5 for AED-exposed children with language delay at 18 months and week 4.3 for AED-exposed children with language delay at 36 months. Mothers of AED-exposed children without language delay most often started supplementation 3 weeks before conception (*p* = 0.01 for 18 months and *p* = 0.05 for 36 months) (figure 2). When we analyzed supplementation intake in different gestational weeks, the proportion using folic acid before the start of the pregnancy and during the first trimester was higher for AED-exposed children without language delay than in children with delay (figure 2).

The interaction between periconceptional folic acid use and AED exposure after adjustment for relevant covariates was significant for global language score at 18 months (*p* = 0.04) and both global and expressive language score at 36 months (*p* < 0.001 and *p* = 0.01, respectively) (data available from Dryad, figure 1, doi.org/10.5061/dryad.1237b6m). In AED-exposed children, the AR of no periconceptional folic acid intake was 0.51 for global language delay at 18 months and 0.52 at 36 months and 0.71 for expressive language delay at 36 months without adjustment for covariates. In children of mothers without epilepsy, the corresponding ARs were 0.06, 0.30, and 0.36. The ARs were similar after adjustment for relevant covariates (data available from Dryad, table 5).There was no significant relationship between language score and maternal plasma folate concentrations (data available from Dryad, table 6 and figure 2A) or folic acid dose (≥1 mg [n = 84] vs 0.4 mg [n = 55]) (data not shown) for AED-exposed children. Sensitivity analyses were done with the mothers using valproate or AED polytherapy excluded from the calculations. However, the effects of folic acid on AED-related language delay were similar or strengthened (data available from Dryad, table 7).

### AED concentration and language delay

Higher maternal plasma valproate concentration was significantly correlated with a lower global language score at age 18 months (*r* = −0.50, *p* = 0.04) (data available from Dryad, figure 2, B and C, doi.org/10.5061/dryad.1237b6m). No other significant correlations between language score and maternal or umbilical cord AED concentrations were found (data available from Dryad, table 8 and figure 2, A and C).

## Discussion

We found that in AED-exposed children maternal periconceptional folic acid supplementation was associated with better language outcome compared to children of mothers not using folic acid in the periconceptional period. The apparent protective effect of periconceptional folic acid supplementation was striking in the AED-exposed children compared to the AED-unexposed children of mothers with epilepsy and to children of mothers without epilepsy. For all language outcomes, the adjusted ORs for language delay were lower for AED-exposed children when folic acid supplementation was used compared to no supplementation. The interaction analysis between AED exposure and periconceptional folic acid use showed a synergistic effect on the degree of language delay: no folic acid supplementation had more consequences for language scores in AED-exposed children than in children with no AED exposure. The AR of no folic acid supplementation on language delay was >50% in AED-exposed children, whereas it was of modest importance in the control group.

Our results showing the importance of folic acid for language development are in line with 2 studies that found higher mean verbal index scores at 3 years and higher mean IQ at 6 years age in AED-exposed children of periconceptionally folic acid–supplemented mothers vs those without such supplementation.^9,10^ We have recently found that periconceptional folic acid supplementation and plasma folate status in pregnancy also were associated with fewer autistic traits in AED-exposed children from the same epilepsy cohort.^13^ A modest effect of folic acid supplementation on risk of autism was also seen in children of mothers without epilepsy.^8^ However, other studies did not find an association between folic acid supplementation and child IQ,^12^ verbal comprehensive intelligence,^11^ or general language function in AED-exposed children.^31,32^ The discrepancy could be due to type of AED exposure and the timing or dose of folic acid supplementation. In addition, different folic acid food fortification practices between countries could blur the association between folic acid supplement and language outcome. Although there is some overlap between autism and language delay, language delay is multifactorial, complex, and much more common than autism.^33–35^ We thus believe only a minor amount of the language delay found in our study might have been attributed to autistic traits.

We found that the critical period for maternal folic acid supplementation to prevent language delay in AED-exposed children was from 4 weeks before the start of the pregnancy and until the end of the first trimester. There was no significant association between language delay and folic acid supplementation later in pregnancy. Previous studies in the general population similarly highlight the periconceptional period for folic acid supplementation to prevent language delay.^3,6^

The larger proportion of language delay in lamotrigine-exposed children with no folic acid supplementation compared to those with supplementation has not been reported previously. However, mean IQ was higher in lamotrigine-exposed children who had been supplemented with folic acid compared to those who had not.^9^ In rodents given lamotrigine, folic acid supplementation improved their epilepsy, mood, and memory.^36^ Low serum folate concentrations have been reported after lamotrigine therapy.^37^ Impaired neurodevelopment after lamotrigine exposure in utero has been discussed, but data have been conflicting.^38,39^ A particularly beneficial effect of periconceptional folic acid supplementation on language function in lamotrigine-exposed children is possible and could explain previous discordant results.

We did not find any correlation between folic acid doses or plasma folate concentrations and language delay. The maternal plasma samples were obtained during gestational week 17 to 19, which may not reflect accurately the folic acid supplement use reported before and very early in the pregnancy.^40^ The exact dose of folic acid recommended to women with epilepsy who use AEDs has not been established.^2^ The safety of high-dose folic acid supplement use in women with epilepsy and in the general population is still debated.^41,42^ Folic acid dose recommendations cannot yet be specified for individual AEDs, although several AEDs interact with folic acid metabolism.^4,5,37^

We found a correlation between high maternal plasma valproate concentrations and low language score in children 18 months of age. This is in line with previous data showing a dose-dependent increased risk of language delay after valproate exposure in utero.^38,43,44^ Maternal drug dose has been used as a proxy for child exposure, but valproate use in women of childbearing age has shown an extensive interindividual pharmacokinetic variability, with dose being a poor reflector of concentration.^45^

Strengths of our study are a large data collection including 2 different epilepsy groups. Both the maternal diagnosis of epilepsy and the type of AEDs have been validated. Maternal plasma folate and AED concentrations in umbilical cord and maternal blood were measured. Selection bias in the MoBa is moderate and does not affect exposure-outcome association analysis.^46^ We adjusted for relevant confounders. Sensitivity analyses confirmed that the association of no use of folic acid with delayed language was not confounded by the frequency of polytherapy or valproate users. Our data have been obtained from parental reporting, and the interobserver reliability between parents and professional examiners for ASQ has been validated as high.^24^ Parents are good evaluators of language abilities of their children.^47^

Weaknesses of our study include relatively low numbers of children exposed to specific AEDs and different doses of folic acid. This limits the interpretation of folate effects linked to individual AEDs and the effects of AED concentrations on language development; both are areas for future research. There were some loss to follow-up at 18 and 36 months of age. We do not have data on language development in the nonresponding group and do not know whether language delay in the child influenced the mother's motivation for continued participation. None of the children were assessed blindly because the language delay relied on maternal report only, not on a formal neuropsychologist review. Although the participants were included from 1999 to 2008 when less was known about the potential harmful effects of AEDs on language development, mothers who used AEDs during the pregnancy might have been more vigilant when reporting language skills than mothers with epilepsy not using AEDs. The mothers reported folic acid use before pregnancy during gestational week 17 to 19, and this may have an effect on the accuracy of these estimates. We do not have data on parental IQ or familial risk of language delay and therefore could not adjust for these factors in our analyses. Plasma folate concentrations were not measured at the most critical point for child development. The lack of mandatory folic acid fortification in Norway may have accentuated our results. Thus, our findings may not be generalizable to countries with a mandatory folic acid food fortification practice.

We found an apparent extensive protective effect of maternal folic acid supplementation from 4 weeks before the start of the pregnancy and during the first trimester on language delay at age 18 and 36 months in AED-exposed children of mothers with epilepsy. This effect was much stronger in AED-exposed children compared to children of mothers without epilepsy because no folic acid supplementation had more consequences for language scores in AED-exposed children compared to children not exposed to AEDs. From these findings, we advocate daily folic acid intake in all women on AEDs who are likely to become pregnant to decrease the risk of AED-mediated language delay.

## Glossary

AED: antiepileptic drug
AR: attributable risk
ASQ: Ages and Stages Questionnaires
CI: confidence interval
hmTHF: 4-alfa-hydroxy-5-methyltetrahydrofolate
MBRN: Medical Birth Registry of Norway
MoBa: Norwegian Mother and Child Cohort Study
mTHF: 5-methyltetrahydrofolate
OR: odds ratio
RR: relative risk
